# Physiological and RNA sequencing data of white lupin plants grown under Fe and P deficiency

**DOI:** 10.1016/j.dib.2019.104069

**Published:** 2019-05-28

**Authors:** Laura Zanin, Silvia Venuti, Fabio Marroni, Alessandro Franco, Michele Morgante, Roberto Pinton, Nicola Tomasi

**Affiliations:** Dipartimento di Scienze Agroalimentari, Ambientali e Animali, University of Udine, via delle Scienze 206, I-33100, Udine, Italy

**Keywords:** Fe acquisition, Nutrient transporters, P acquisition, Plant nutrition, RNA-seq, Root transcriptome, Root uptake, Strategy-I

## Abstract

This DIB article provides details about transcriptional and physiological response of Fe- and P-deficient white lupin roots, an extensive and complete description of plant response is shown in the research article “Physiological and transcriptomic data highlight common features between iron and phosphorus acquisition mechanisms in white lupin roots” Venuti et al. [1].

White lupin plants were grown under hydroponic system and three different nutritional regimes: Fe deficiency (-Fe), P deficiency (-P), or Fe and P sufficiency (+P + Fe).

Depending on nutritional treatment, white lupin plants showed changes in the fresh weights, in root external acidification and Fe^III^-reductase activity. Moreover, the transcriptomic changes occurring in apices and clusters of Fe-deficient lupin roots were investigated and compared with differences of gene expression occurring in P-deficient plants (-P) and in Fe- and P-sufficient plants (+P + Fe). Transcriptomic data are available in the public repository Gene Expression Omnibus (http://www.ncbi.nlm.nih.gov/geo) under the series entry (GSE112220). The annotation, mapping and enrichment analyses of differentially modulated transcripts were assessed.

**Table:** Specifications table

Subject area	Plant science and mineral nutrition
More specific subject area	Transcriptional and physiological plant response to nutritional deficiency
Type of data	Images (shoot and root morphology, root acidification activity, root Fe^III^-reductase activity), graphs (enrichment analyses, metabolism overview of transcriptomic changes), Tables
How data was acquired	RNA sequence data (50-bp single end reads) was obtained with an Illumina Hiseq2000 platform.
Data format	Analysed data
Experimental factors	White lupin plants were grown for 32 days under 3 different nutritional regimes: Fe deficiency (-Fe), P deficiency (-P), or Fe and P sufficiency (+P + Fe)
Experimental features	The whole root system of white lupin plants was placed in contact with agarose gel containing pH indicator bromocresol purple (that allowed the measure of acidification activity), or containing BPDS, (a chelating agent of Fe^II^ used to evaluate the root Fe^III^-reductase activity). Beside morphometric and physiological evaluation the white lupin roots were sampled (dividing root structures: apices and cluster roots). mRNA was isolated from samples and sequenced by Illumina Hiseq2000.
Data source location	Plant growth and analyses were performed under controlled conditions at the Dipartimento di Scienze Agroalimentari, Ambientali e Animali (University of Udine, via delle Scienze 206, I-33100 Udine, Italy) and the RNA sequencing analyses were performed at the Institute of Applied Genomics (IGA, Udine, Italy)
Data accessibility	Raw sequencing data are in the NCBI public repository, the series entry is GSE112220: platform GPL23437 and 12 samples (GSM3061077; GSM3061078; GSM3061079; GSM3061080; GSM3061081; GSM3061082; GSM3061083; GSM3061084; GSM3061085; GSM3061086; GSM3061087; GSM3061088) under the BioProject number PRJNA445290 (http://www.ncbi.nlm.nih.gov/geo).
Related research article	S. Venuti, L. Zanin, F. Marroni, A. Franco, M. Morgante, R. Pinton, N. Tomasi, Physiological and transcriptomic data highlight common features between iron and phosphorus acquisition mechanisms in white lupin roots, Plant Sci., 285, 2019, 110–121 [1].

**Table:** 

**Value of the data** • The Fe deficient response of white lupin plants was analysed through root physiological and transcriptomic analyses • Under Fe deficiency, morphological changes occurred at the root system with the formation of cluster roots, quite similar to those occurring under P deficiency • The transcriptomic study was performed on apices and on cluster roots of Fe-deficient plants, allowing a spatial resolution of Fe-deficient response • Similarities and differences were highlighted comparing the transcriptional changes of Fe deficient cluster roots with those occurring in P-deficient cluster roots

## 1 Data

**Fig. 1:**
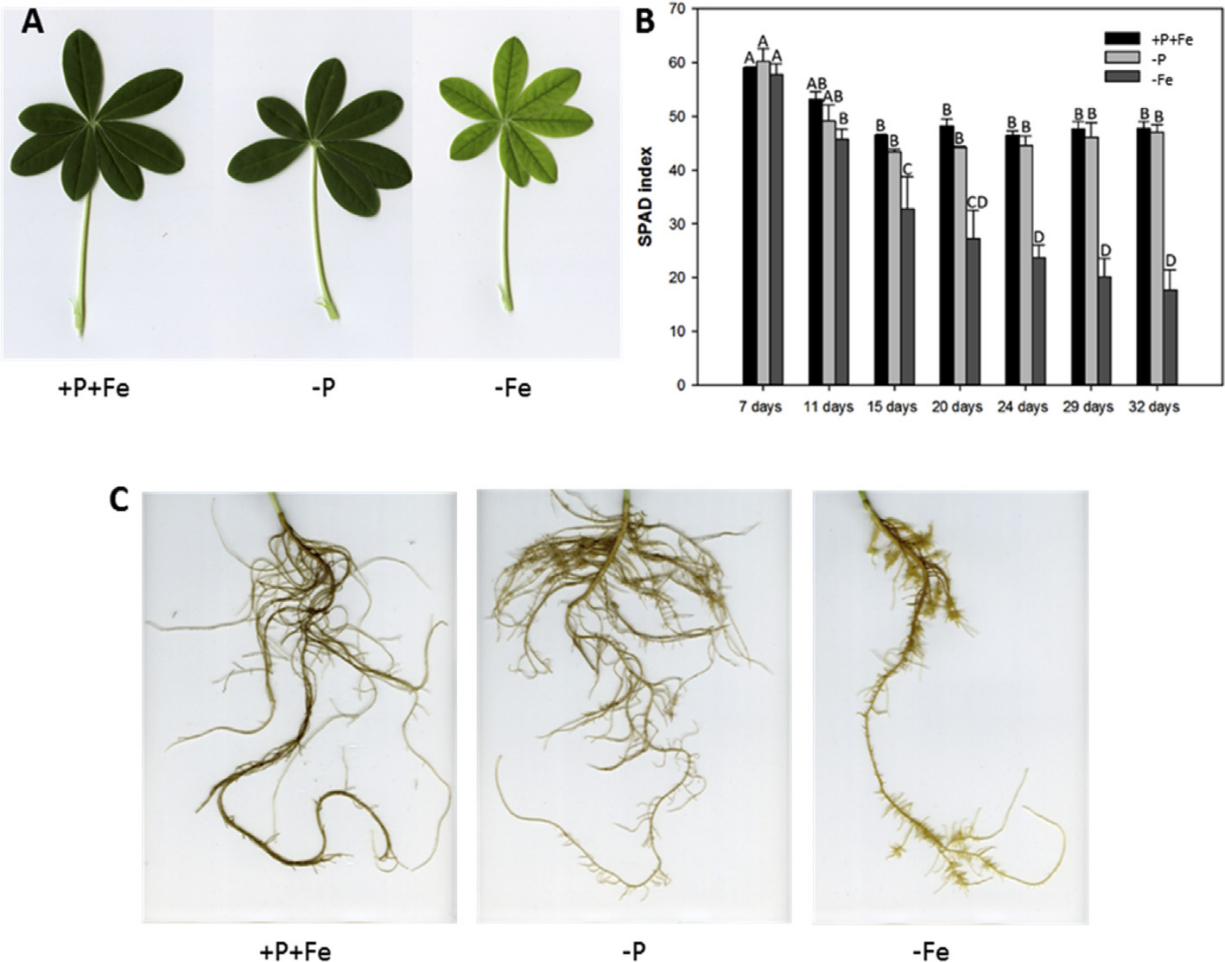
Shoot and root apparatus of lupin plants grown under complete nutrient solution (+P + Fe), or under P deficiency (-P) or Fe deficiency (-Fe). **A**, shoots of +P + Fe, -P and -Fe plants, respectively (from left to right). **B**, SPAD index values of leaf tissues were measured at the beginning of the treatment (7-day old) and up to 25 days of treatment (32-day old) lupin plants grown under different nutritional conditions. Data are means +SD of three independent experiments (capital letters refer to statistically significant differences among the mean, ANOVA: Holm–Sidak method, N = 3, p-value < 0.05). **C**, root systems of +P + Fe, -P and -Fe plants, respectively (from left to right).

**Fig. 2:**
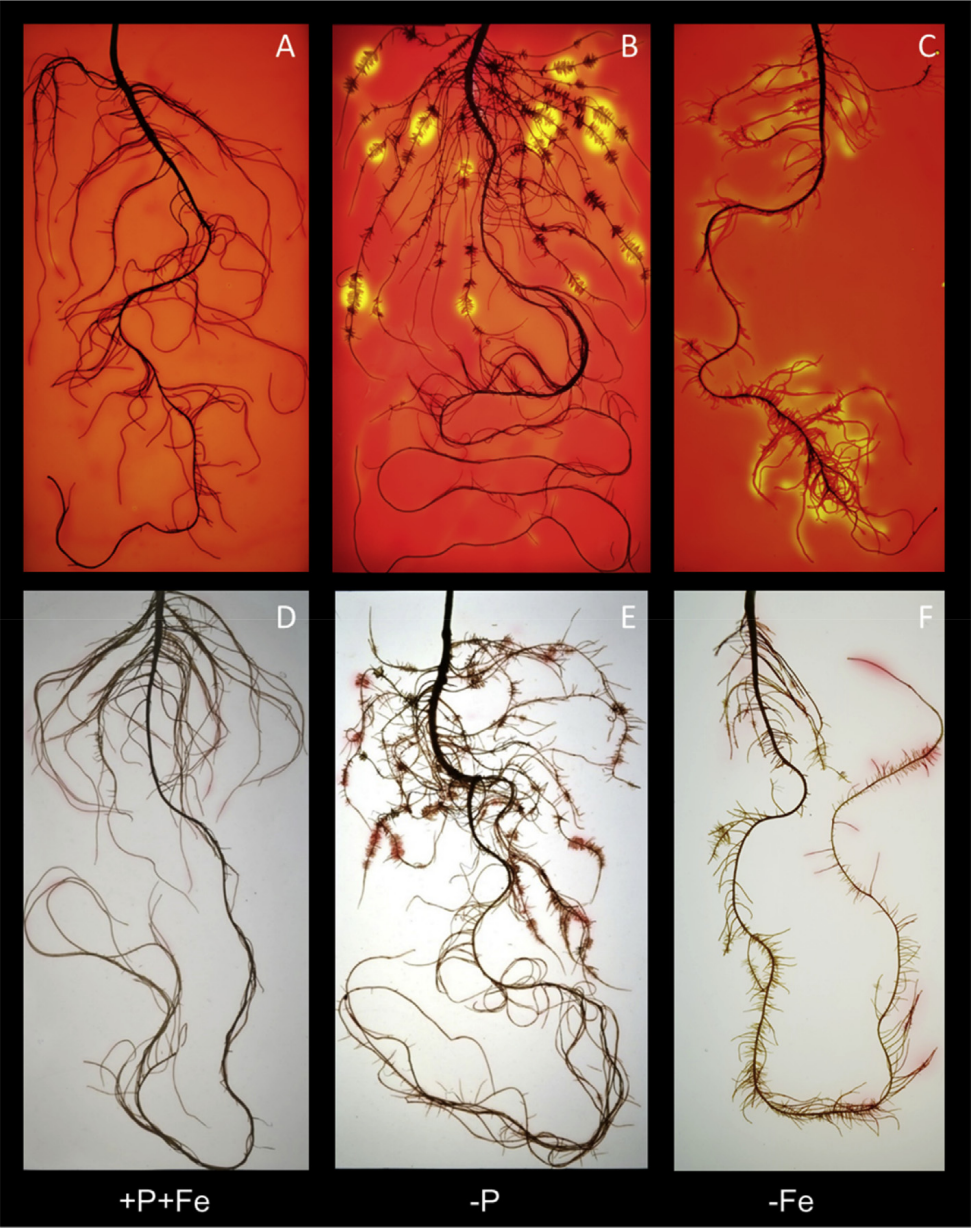
In the above panels, the external acidification by roots of lupin plants grown under control condition (+P + Fe; **A**), P deficiency (-P, **B**) or Fe deficiency (-Fe, **C**; panels above) is shown. Roots were imbedded for 4 hours in agar gel containing pH indicator (Bromocresol purple); yellow indicates acidification of agar gel (pH < 5.5) and purple indicates an alkalization above pH 7. In the below panels, the visualization of Fe(III)-reduction by roots of lupin plants grown under control condition (+P + Fe; **A**), P deficiency (-P, **B**) or Fe deficiency (-Fe, **C**) is shown. The roots (32-day-old plants) were placed for 6 hour in agar gel containing 100 μM Fe (III)-EDTA and 300 μM BPDS; the formation of Fe(II)-BPDS determines the reddish color.

**Fig. 3:**
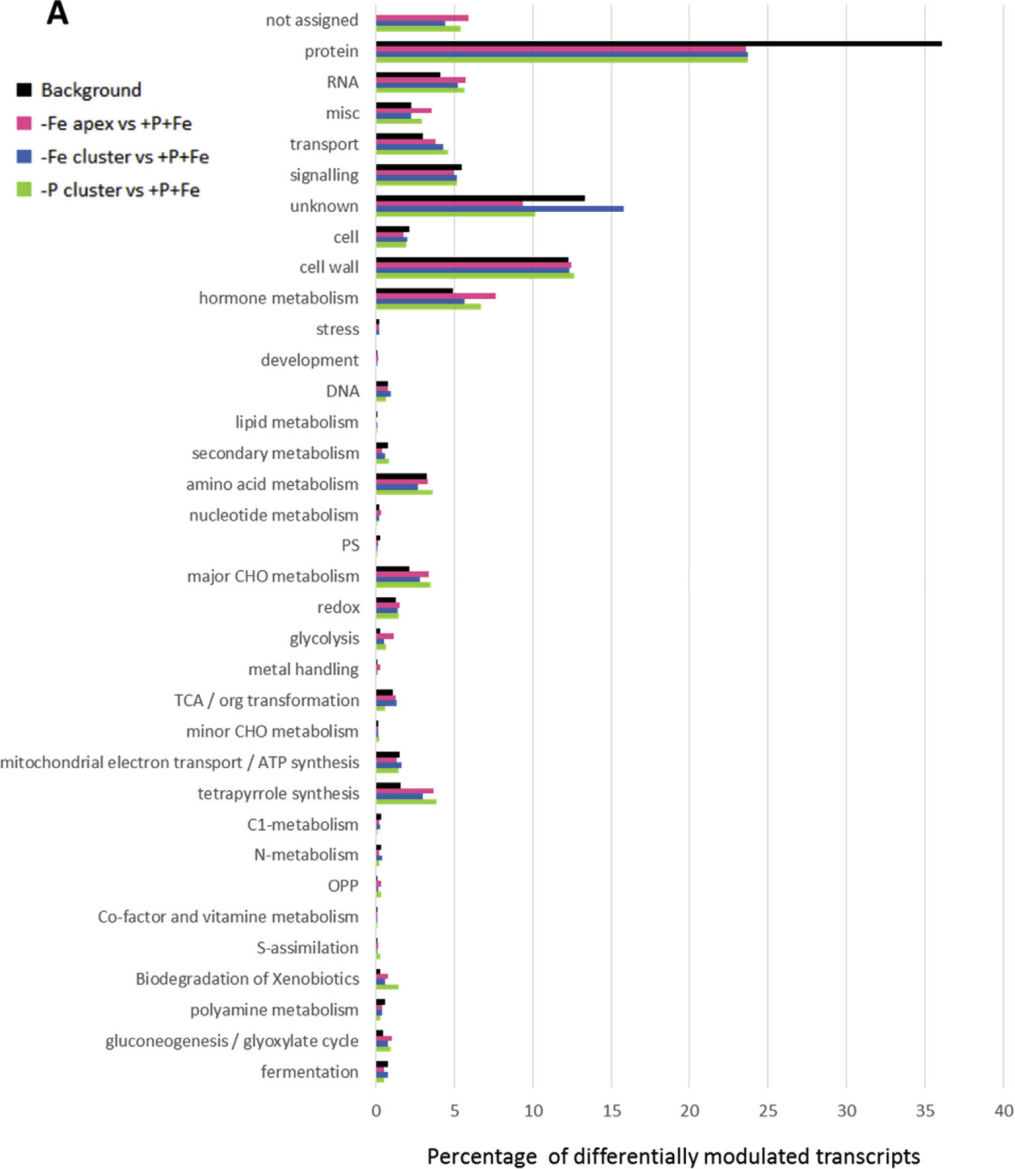

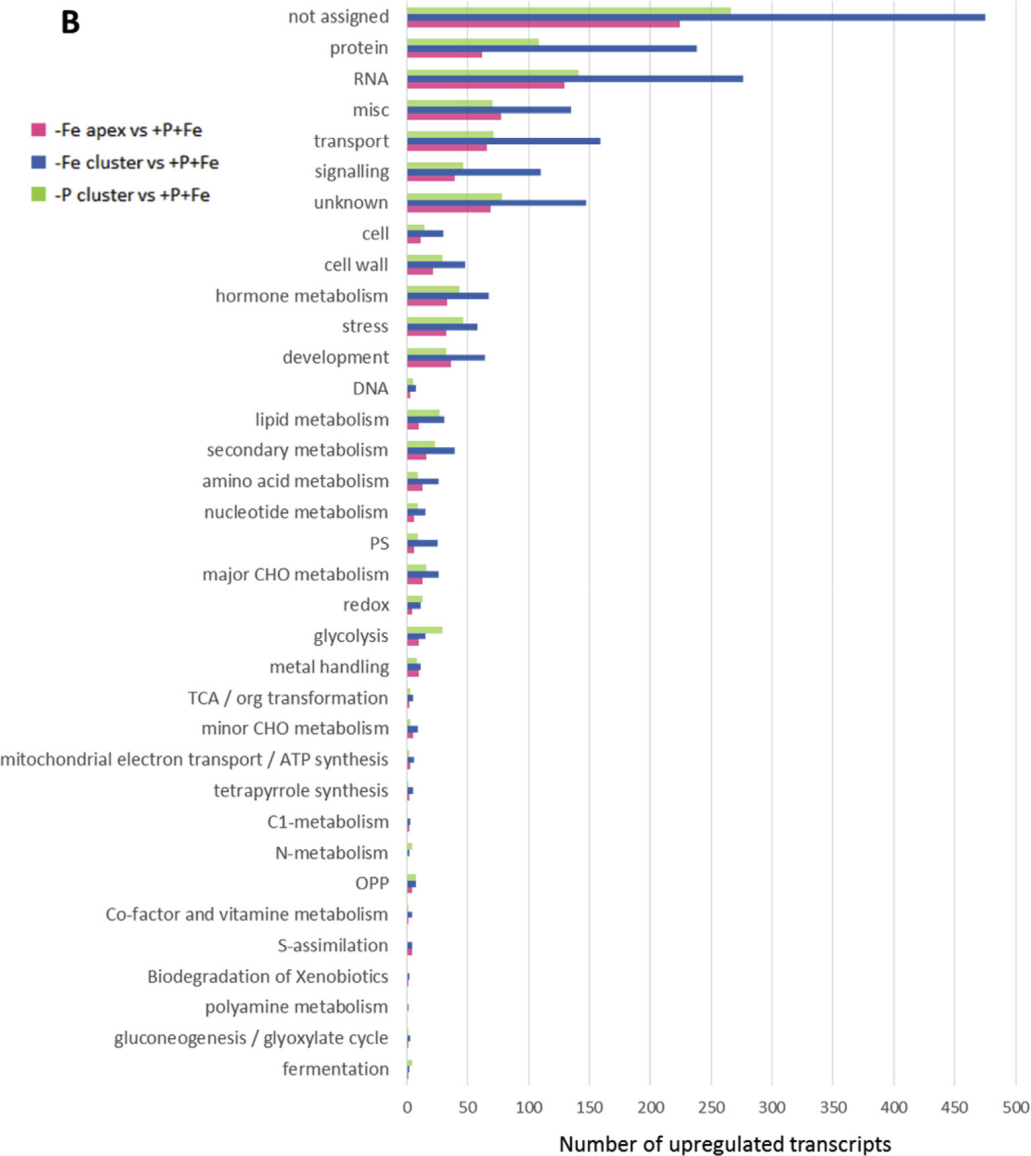

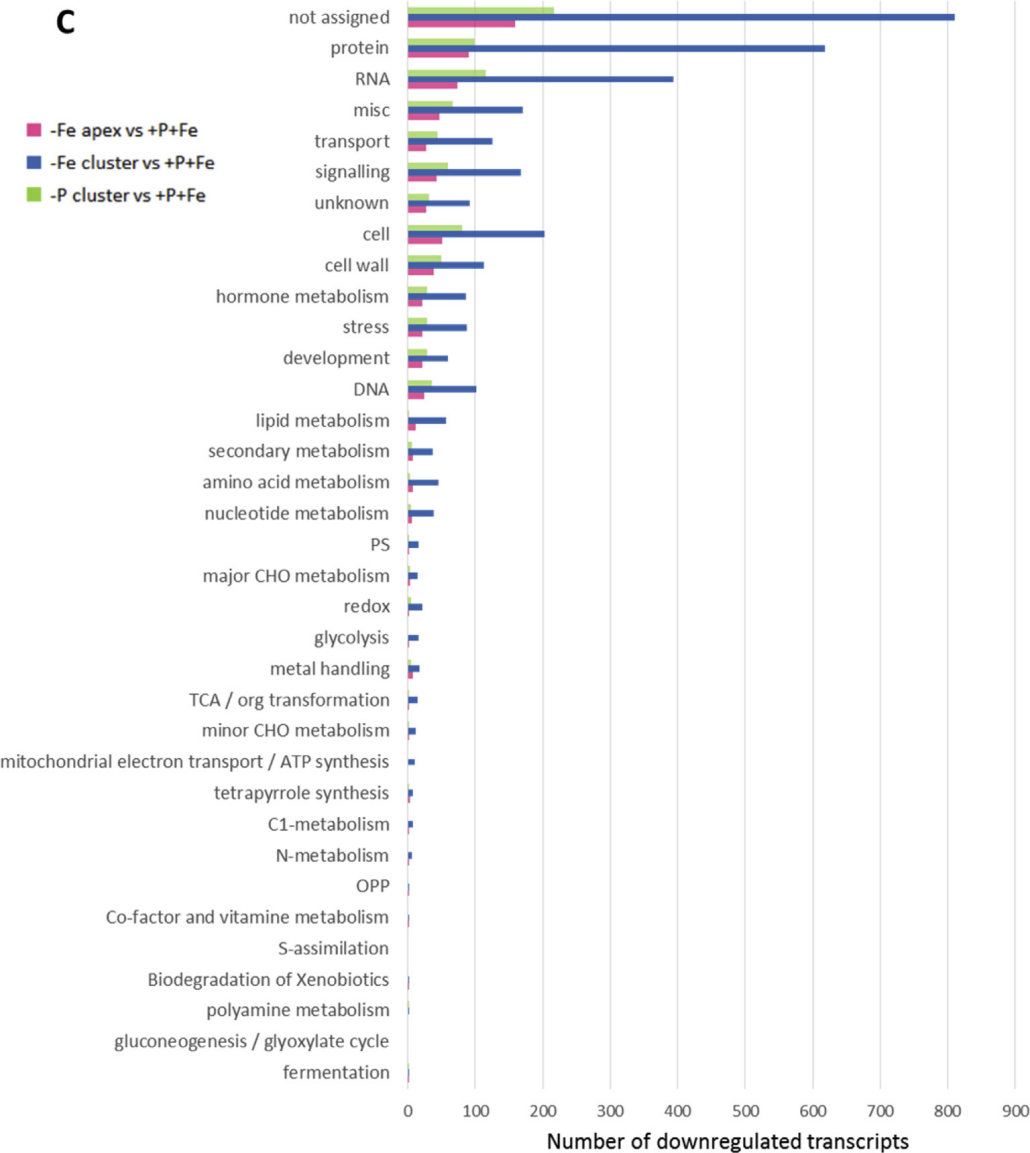
Enrichment of MapMan-bins of the significantly regulated genes in three comparisons: Fe apex vs + P + Fe, -Fe cluster vs + P + Fe and -P cluster vs + P + Fe and the background (*Gmax_189,*https://mapman.gabipd.org/) used as reference annotated transcriptome. The enrichment of each MapMan bin is expressed as percentages of transcripts (**A**); graphical indication of the number of upregulated (**B**) and downregulated (**C**) transcripts is also shown.

**Fig. 4:**
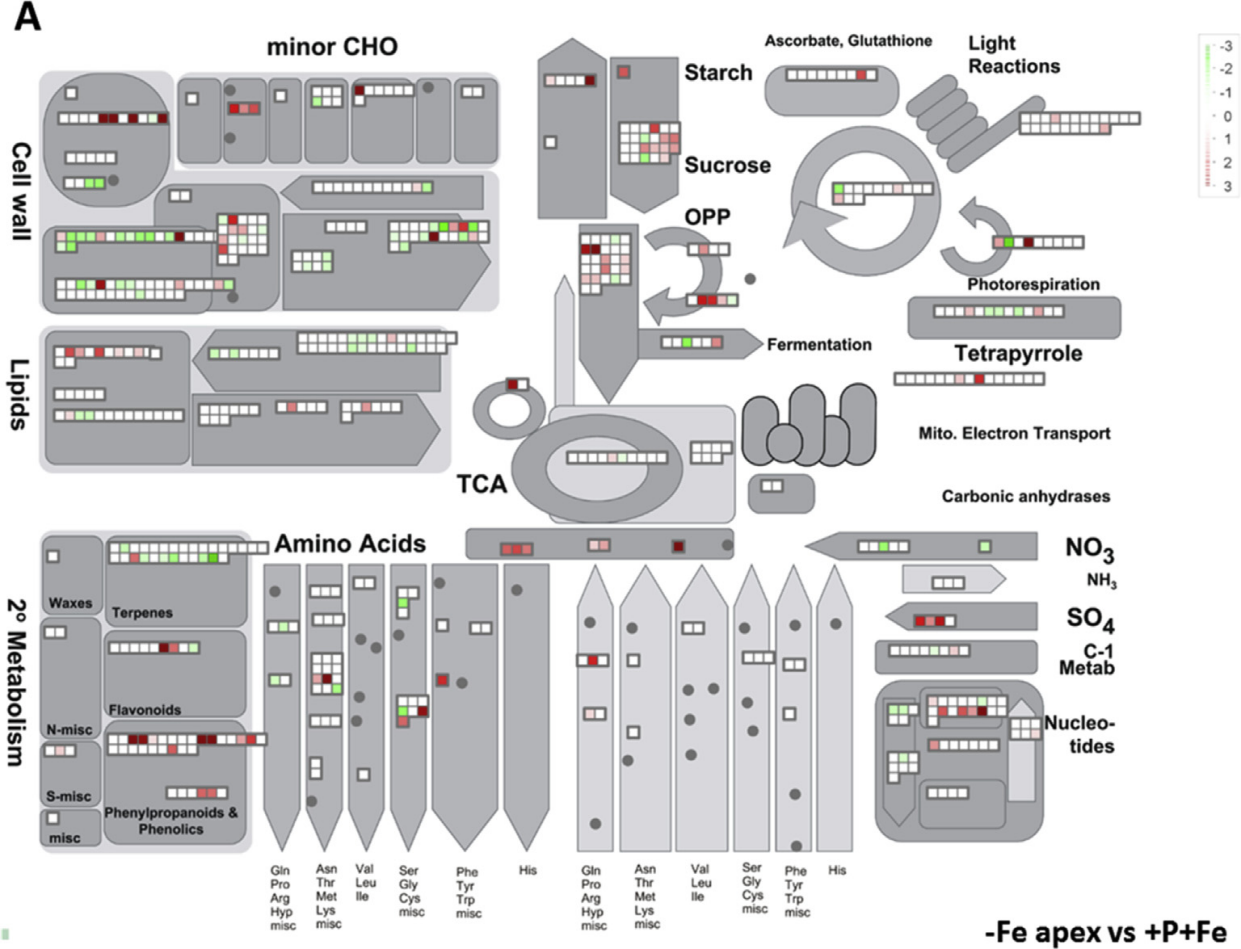

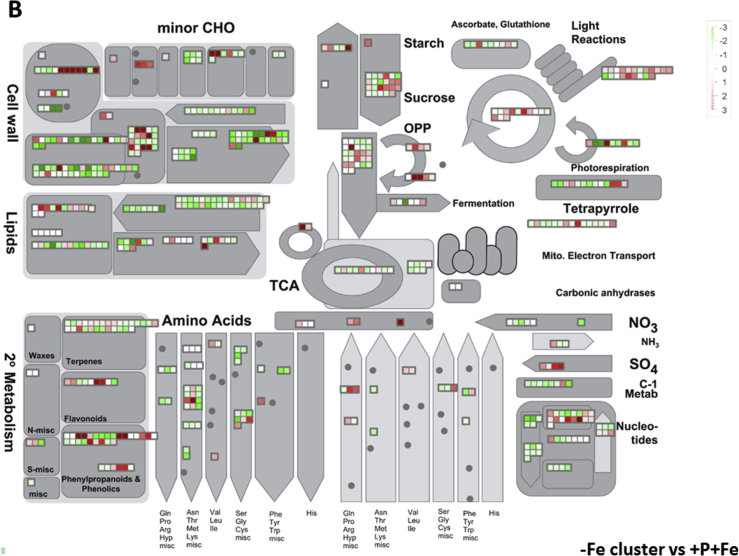

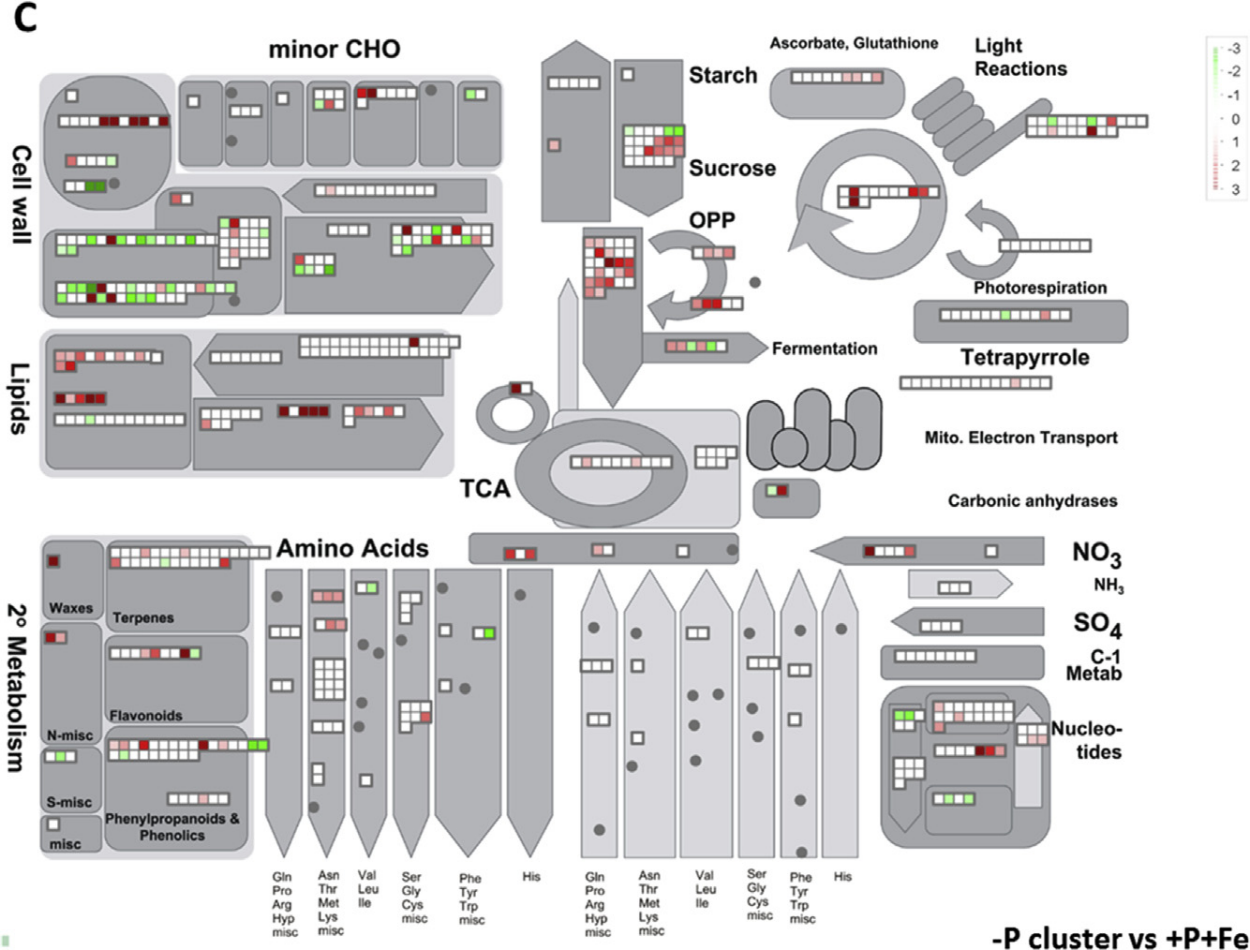
Mapping of transcriptional modulation of genes involved in the cell metabolism in the three transcriptomic comparisons: Fe apex vs + P + Fe (**A**), -Fe cluster vs + P + Fe (**B**) and -P cluster vs + P + Fe (**C**). Color scale refers to the Log_2_ FC values of differentially expressed transcripts: red color refers to those transcripts positively regulated by treatment, while in green are transcripts negatively regulated.

**Supplementary figure S1:**
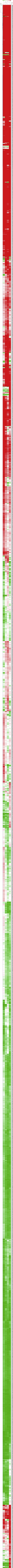
Hierarchical clustering analysis of four transcriptomic profiles: +P + Fe, -Fe apex, -Fe cluster and -P cluster of lupin roots, output from TMeV software (defaults parameters were used with distance metric: Euclidean distance, linkage method: complete; N = 3, q-value <0.05). From green (low values) to red (high values), color scale refers to the FPKM values of transcripts.

In this data article, physiological and transcriptomic analyses of Fe-deficient and P-deficient white lupin plants are reported. At the end of the treatment (32 days), fresh and dry weights (FW and DW) of white lupin were recorded (Table 1). Visible symptoms of nutritional regimes were observed in lupin plants, as the interveinal chlorosis in -Fe leaves and a dark blue-green color in -P leaves. Under both nutritional conditions, -Fe and -P plants showed the occurrence of cluster roots at the root system (Fig. 1). Moreover the root system of intact plants was placed in contact on agarose gel containing a pH indicator (bromocresol purple, useful to evaluate the acidification of root external solution) and on agarose gel containing BPDS (for the evaluation of the root Fe^III^-reductase activity; Fig. 2). To evaluate the plant response also at molecular level, transcriptomic analyses were performed through RNA sequencing technology (50-bp single end reads obtained using an Illumina Hiseq2000 platform). Alignment against the transcriptome of *Lupinus albus* Gene Index Version 2 (LAGI02) [2] was performed with TopHat version 2.0.5 [3] with default parameters. Further details on RNA-sequencing analyses are reported in Venuti et al. [1]. The transcriptomic profiles of apices and cluster roots of Fe-deficient and P-deficient plants were obtained and a hierarchical clustering analysis of four profiles was performed by MeV software v.4.8 (http://mev.tm4.org/, Supplementary Figure S1). The four profiles were compared against the +P + Fe profile obtaining three comparisons, as “–Fe apex vs + P + Fe”, “-Fe cluster vs + P + Fe” and “-P cluster vs + P + Fe”. The differentially modulated transcripts were annotated using *Glycine max* as reference (Gmax_189_annotation_info.txt annotation file is available on ftp://ftp.jgi-psf.org/pub/compgen/phytozome/v9.0/Gmax/) and classified into hierarchical categories ('BINs') using MapMan software tool (Version 3.6.0RC1) [4]. For each hierarchical categories, an enrichment analyses of differentially modulated transcripts was performed and expressed as percentages of modulated transcripts (Fig. 3A) or as number of upregulated (Fig. 3B) and downregulated (Fig. 3C) transcripts. To evaluate the number of commonly modulated transcripts among three transcriptomic comparisons, a Venn diagram was performed (Figure 5 in Venuti et al. [1]), in Supplementary Table S1, the differentially modulated transcripts are clustered according to the Venn diagram regions and to the hierarchical categories (‘BINs'). For each comparison the differentially modulated transcripts were also mapped by MapMan software providing a metabolic overview of transcriptional changes (Fig. 4).

**Table 1:** Fresh weight (FW) and dry weight (DW) of lupin plants under three nutritional treatments: control (+P + Fe), P deficiency (-P) or Fe deficiency (-Fe). Data are means +SD of three independent experiments (capital letters refer to statistically significant differences among the mean, ns: no significant statistical difference, ANOVA Holm–Sidak, N = 3, p-value < 0.05).

	Fresh weight (g)	Dry weight (g)
Leaves:		
+P + Fe	6.28 ± 0.92 A	0.49 ± 0.06 ns
-P	4.93 ± 0.97 AB	0.41 ± 0.11 ns
-Fe	3.81 ± 0.63 B	0.32 ± 0.02 ns
Roots:		
+P + Fe	2.29 ± 0.30 A	0.14 ± 0.04 ns
-P	2.18 ± 0.21 A	0.13 ± 0.04 ns
-Fe	1.29 ± 0.07 B	0.10 ± 0.01 ns

### 1.1 Experimental design, materials and methods

The capability of white lupin roots to acidify the external media was visualized on agar gel (0.9% w/v agar layer containing 0.04% w/v bromocresol purple, as pH indicator) as previously described by Zanin et al. [5]. The Fe(III)-reductase activity of white lupin roots was evaluated on agarose gel containing the bathophenanthroline-disulfonate (BPDS). For further details on experimental set up and methods see the research article [1].

## Appendix A Supplementary data

Supplementary table S1 Number of differentially modulated transcripts referring to different regions of Venn diagram (Fig. 5) and annotated accordingly to MapMan bin categories (*Gmax_189* annotated transcriptome, https://mapman.gabipd.org/). Supplementary table S1
