# Supplementary material for: Physiological and RNA sequencing data of white lupin plants grown under Fe and P deficiency
Source: Data Brief. 2019 May 28;25:104069. doi: 10.1016/j.dib.2019.104069 (PMC6562269; doi:10.1016/j.dib.2019.104069)
Supplement: Supplementary file 1 — Multimedia component 1 [file mmc1.pdf]

Conflict of Interest and Authorship Conformation Form

Please check the following as appropriate:

- ☑ All authors have participated in (a) conception and design, or analysis and interpretation of the data; (b) drafting the article or revising it critically for important intellectual content; and (c) approval of the final version.

- ☑ This manuscript has not been submitted to, nor is under review at, another journal or other publishing venue.

- ☑ The authors have no affiliation with any organization with a direct or indirect financial interest in the subject matter discussed in the manuscript

- ☑ The following authors have affiliations with organizations with direct or indirect financial interest in the subject matter discussed in the manuscript:

| Author's name | Affiliation |
| --- | --- |
| | |

Udine, 20th of May, 2019

Laura Zanin
